# Antiproliferative effects of mesenchymal stem cells carrying *Newcastle disease virus* and *Lactobacillus Casei* extract on CT26 Cell line: synergistic effects in cancer therapy

**DOI:** 10.1186/s13027-023-00521-y

**Published:** 2023-07-31

**Authors:** Akbar Ghorbani Alvanegh, Majid Mirzaei Nodooshan, Ruhollah Dorostkar, Reza Ranjbar, Bahman Jalali Kondori, Alireza Shahriary, Karim Parastouei, Soheil Vazifedust, Elmira Afrasiab, Hadi Esmaeili Gouvarchinghaleh

**Affiliations:** 1 Human Genetics Research Center, Baqiyatallah University of Medical Sciences, Tehran, Iran; 2 Applied Virology Research Center, Baqiyatallah University of Medical Sciences, Tehran, Iran; 3 Molecular Biology Research Center, Systems Biology and Poisonings Institute, Baqiyatallah University of Medical Sciences, Tehran, Iran; 4 Department of Anatomical Sciences, Faculty of Medicine, Baqiyatallah University of Medical Sciences, Tehran, Iran; 5 Baqiyatallah Research Center for Gastroenterology and Liver Diseases (BRCGL), Baqiyatallah University of Medical Sciences, Tehran, Iran; 6 Chemical Injuries Research Center, Systems Biology and Poisonings Institute, Baqiyatallah University of Medical Sciences, Tehran, Iran; 7 Health Research Center, Life Style Institute, Baqiyatallah University of Medical Sciences, Tehran, Iran; 8 Department of Medical Biotechnology, National Institute of Genetic Engineering and Biotechnology (NIGEB), Tehran, Iran

**Keywords:** Colorectal cancer, Mesenchymal stem cells, *Newcastle disease virus*, *Lactobacillus casei*

## Abstract

**Background and aims:**

Colorectal Cancer (CRC) is a frequent malignancy with a high mortality rate. Specific inherited and environmental influences can affect CRC. Oncolytic viruses and bacteria in treating CRC are one of the innovative therapeutic options. This study aims to determine whether mesenchymal stem cells (MSCs) infected with the *Newcastle Disease Virus* (NDV) in combination with *Lactobacillus casei* extract (L. casei) have a synergistic effects on CRC cell line growth.

**Materials and methods:**

MSCs taken from the bone marrow of BALB/c mice and were infected with the 20 MOI of NDV. Then, using the CT26 cell line in various groups as a single and combined treatment, the anticancer potential of MSCs containing the NDV and *L. casei* extract was examined. The evaluations considered the CT26 survival and the rate at which LDH, ROS, and levels of caspases eight and nine were produced following various treatments.

**Results:**

NDV, MSCs-NDV, and *L. casei* in alone or combined treatment significantly increased apoptosis percent, LDH, and ROS production compared with the control group (*P*˂0.05). Also, NDV, in free or capsulated in MSCs, had anticancer effects, but in capsulated form, it had a delay compared with free NDV. The findings proved that *L. casei* primarily stimulates the extrinsic pathway, while NDV therapy promotes apoptosis through the activation of both intrinsic and extrinsic apoptosis pathways.

**Conclusions:**

The results suggest that MSCs carrying oncolytic NDV in combination with *L. casei* extract as a potentially effective strategy for cancer immunotherapy by promoting the generation of LDH, ROS, and apoptosis in the microenvironment of the CT26 cell line.

## Introduction

CRC is the third leading cause of cancer worldwide, and the incidence is rising in developing countries [1]. CRC is a type of cancer known as colorectal adenocarcinoma, which emerges from glandular, large intestine epithelial cells [2]. Cancer occurs when specific epithelial cells undergo genetic or epigenetic changes that give them a selective advantage [3]. These hyper-proliferative cells produce benign adenomas, which may develop into carcinoma and spread over many years due to unusually elevated replication and survival [4]. The survival rates have increased due to improvements in primary and adjuvant therapies for CRC [5]. Surgical resection and chemotherapy, radiation, or biological treatments are options for CRC to remove the tumor and any metastases [6, 7]. Early diagnosis and detection are critical to treating CRC cancer and preventing recurrence [7]. Multiple-agent regimens including one or more drugs, such as Oxaliplatin (OX), Irinotecan (IRI), and Capecitabine (CAP or XELODA or XEL), are currently available, as are single-agent therapies, notably Fluoropyrimidine (5-FU) [8]. When used alone or in conjunction with radiotherapy, chemotherapy is considered the most efficient and popular modality for treating cancer [9]. Scientists are constantly researching better cancer treatment methods [10]. One of the novel cancer immunotherapy strategies is oncolytic viruses and bacteria [11]. Oncolytic viruses that naturally arise or are genetically created to reproduce preferentially in tumor cells and prevent tumor development [12]. These oncolytic viruses have recently been regarded as a successful anticancer therapy [13]. They primarily function by eliminating cancer cells directly, engaging the immune system to fight cancer, and expressing exogenous effector genes [14]. Combined with other treatments like radiotherapy, chemotherapy, and immunotherapy, their multifunctional properties suggest promising application prospects as cancer medicines [15]. NDV is one of five species of viruses under clinical evaluation as vectors for oncolytic cancer therapy, gene therapy, and immune stimulation [16]. When it has specifically infected tumor cells, the immune response triggered by NDV’s envelope protein and intracellular components can successfully eliminate the tumor without harming healthy cells [17]. NDV sensitivity has been demonstrated in the cells of several human malignancies, including lymphoma, glioblastoma, and liver cancer [18]. Because NDV RNA transcription and translation are independent of cell proliferation, the virus can target tumor stem cells, dormant tumor cells, and vaccinated tumor cells exposed to X-rays [19]. The host immune system poses a significant barrier to the use of naked viruses in cancer virotherapy by decreasing the efficacy of the therapy through complement-mediated antibody-dependent neutralization [20]. Oncolytic virus transmission to metastatic cancer locations and therapeutic impact is constrained by the host’s quick production of neutralizing antibodies [21]. Cell carriers have been suggested as a unique strategy to shield the oncolytic virus from the negative consequences of immune-mediated clearance or neutralization [22]. Also, the effectiveness of the oncolytic virus is increased by using cells having an inherent propensity to move inside the tumor microenvironment to deliver anti-cancer medicines [23, 24]. MSCs have the qualities of a potential delivery system that shields oncolytic viruses from the effects of complement-mediated neutralizing antibodies and has the unique capacity to direct them to inflammatory and tumor development areas [25]. Like other microorganisms that treat cancer, oncolytic bacteria must be safe enough to target therapy and destroy the tumor while preserving the patient’s life [26]. Several bacteria prefer to gather inside tumors, where they function in an oncolytic manner [27]. The preferred tumor replication of *Salmonella*, *Streptococcus*, *Listeria*, *Escherichia*, *Clostridium*, *Bifidobacterium*, *Caulobacter*, *Proteus*, *Lactobacillus*, *Klebsiella*, or *Mycobacterium* has been examined by several organizations over the past few decades [28]. *Lactobacillus casei* (L. casei) is utilized as an acid-producing starting culture in the creation of fermented foods and produces lactic acid as a byproduct of the fermentation of carbohydrates [29]. Improvements in rheumatoid arthritis and stomach microbial balance have all been linked to using *L. casei* as a dietary supplement [30]. Leukemia and liver cancer growth inhibition has anti-cancer properties [31]. This study assessed the synergistic effects of *L. casei* and mesenchymal stem cells (MSCs) infected with *Newcastle disease virus* (NDV) on CRC cell line growth.

## Materials and methods

### Cell culture

The National Cell Bank of Iran provided the CT26.WT (ATCC CRL-2638) cell line (Pasteur Institute of Iran, Tehran). DMEM-containing flasks were seeded with cancerous cells. 10% fetal bovine serum was added to the medium utilized. Atmospheres of 5% CO2 and 37 °C were used to incubate the cells. The attached cells were trypsinized, counted, and put into a 96-well plate, with 1 × 10^4^ cells in each well. The plate was then incubated for 24 h at 37 °C and 5% CO2 to allow the cells to adhere to the bottom of the wells [32].

### Isolation of MSCs and viral infection with NDV

As previously mentioned, the bone marrow-derived MSCs were separated based on their capacity to stick to the culture plates. The sacrificed BALB/c mice had their bone marrow drained from their tibias and femurs. The cells were grown in T25 culture flasks with a DMEM medium supplemented with 15% FBS in a humidified incubator with 5% CO2 at 37 °C after two rounds of washing. The adherent cells were fed twice weekly on the fourth day, while the non-adherent cells were carefully discarded. The MSCs were separated by trypsin/EDTA at 80% confluence (cells were examined for the surface expression of a minimal panel to characterize MSCs at passage three by Flow cytometry), and the isolated MSCs were grown in a 96-well plate for 24 h. The MSCs were infected with the 20 multiplicity of infection (20 MOI) [33] of the LaSota strain *Newcastle disease virus* for one hour in a DMEM medium. Following that, PBS was used to remove free viruses from the supernatant of cells [33]. Then, 10^5^ MSCs-NDV [33] were counted and transferred to 96 plates where CT26 cells were cultured, and after 72 h, evaluations were done.

### Preparation of L. casei extracts

3 × 10^8^ CFU/ml of cultivated *Lactobacillus casei* (ATCC 393) were heated at 56 °C for 60 min to create the bacterial extract, which was then centrifuged for use in the current experiment [34].

### Experimental design

Our university’s ethical committee approved this study’s protocols (approval number: IR.BMSU.REC.1399.507). The CT26 cells were suspended in DMEM, cultivated on a plate, and randomly divided into six groups, including one negative control (NC; untreated cells), four treatment groups, and one positive control using Fluorouracil (5FU) at concentrations of 20 μM, respectively. The characteristic of the treatment and control groups are displayed in Table 1. Single or combined agent treatment groups were established.

**Table 1:** The characteristics of the studied groups

Groups	Abbreviation	Characteristics (in 100 μL of PBS)
Negative Control	NC	PBS
Positive Control	PC	20 μM (5FU)
*Newcastle Disease Virus*	NDV	20 MOI
Mesenchymal stem cell carrying NDV	MSCs-NDV	10^5^ cells/well
*Lactobacillus casei* extract	L. casei	3 × 10^8^ CFU/ml
MSCs-NDV + L. casei + NDV	Combined treatment	same as before

### Cell viability

The MTT assay for the viability of CT26 cells was evaluated against single and combination treatment groups, according to Esmaeili Gouvarchin Ghaleh et al. study (2019) [20]. To sum up, cells in 96-well plates were treated with a single or combined treatment for 72 h. Four hours before the 72-hour period ended, MTT (20 μl, 5 mg/ml) was added to each well. The plate was then incubated at 37 °C for four hours to produce a purple formazan result. Dimethyl sulfoxide (DMSO), 100 μl, was added to the plate, and it was then incubated at 37 °C for 10 min to see if the purple crystals would dissolve. At a wavelength of 570 nm, an ELISA plate reader evaluated each well’s optical density (OD). The following technique was used to determine the cell viability.

$${\rm{cell}}\,{\rm{viability}}\,\left( \% \right)\, = \,\frac{{{\rm{OD}}\,{\rm{of}}\,570\,{\rm{nm}}\,{\rm{of}}\,{\rm{treated}}\,{\rm{cells}}}}{{{\rm{OD}}\,{\rm{of}}\,570\,{\rm{nm}}\,{\rm{of}}\,{\rm{control}}\,{\rm{cells}}}}\, \times \,100$$

### ROS production assay

ROS production was measured using dichlorodihydrofluorescein diacetate. After DCFHDA enters the cell passively, it reacts with ROS to produce the highly Fluorescent Chemical Dichlorofluorescein (DCF). The cells were temporarily treated with a single or combined treatment before being transferred at a rate of 1 × 10^6^ cells per well to 6-well plates for 72 h. The cells were stained with 20 μM DCFH-DA within 30 min and then incubated in DCFH-DA after being washed twice with PBS. After PBS rinsed the cells, the fluorescence intensities were determined by control [35].

### Evaluation of CT26 apoptosis

The Esmaeili Gouvarchin Ghaleh et al. study (2019) examined the apoptosis of CT26 cells using acridine orange and propidium iodide [35]. In conclusion, the cells were treated with a single or combined treatment. PBS was used to rinse the cell solution specific to each group. Then attached cells were trypsinized, and the fluorescent dye (10 μl) was then decanted into the cell pellet in equal parts using acridine orange (10 μg/ml) and propidium iodide (10 μg/ml). Estimation of apoptotic cells (%) was carried out in an updated Neubauer rhodium hem cytometer under fluorescent microscopy (Olympus CKX41).

### Lactate dehydrogenase assay (LDH)

The enzyme lactate dehydrogenase (LDH), widely distributed in the cytosol, transforms lactate into pyruvate. LDH escapes into culture media, and its extracellular level rises when plasma membrane integrity is compromised. The LDH Kit was used to measure cytotoxicity. This assay quantifies the lactate dehydrogenase (LDH) released by injured cells. The cells were treated with a single or combined treatment for 72 h. The culture supernatant was taken after 72 h of treatment and incubated with the reaction mixture. Tetrazolium salt is reduced to Formosan by the LDH-catalyzed conversion, which may be detected at 492 nm absorption [35].

### Examination of caspase-8, and 9 formation

According to the manufacturer’s instructions, and Jabbari et al. study (2018) used colorimetric assay kits (Sigma-Aldrich, St. Louis, MO) to measure the concentrations of caspase-8 and caspase-9 proteins in CT26 cells after they had been exposed to single or combined treatment groups [36].

### Statistical analysis

Mean and Standard Deviation (SD) were reported for quantitative variables. One-Way ANOVA and LSD post hoc tests were used in this study. All analyses were executed with SPSS (ver. 24), and the statistical significance level was considered less than 0.05.

## Results

### MSCs and flow cytometric analysis

**Fig. 1:**
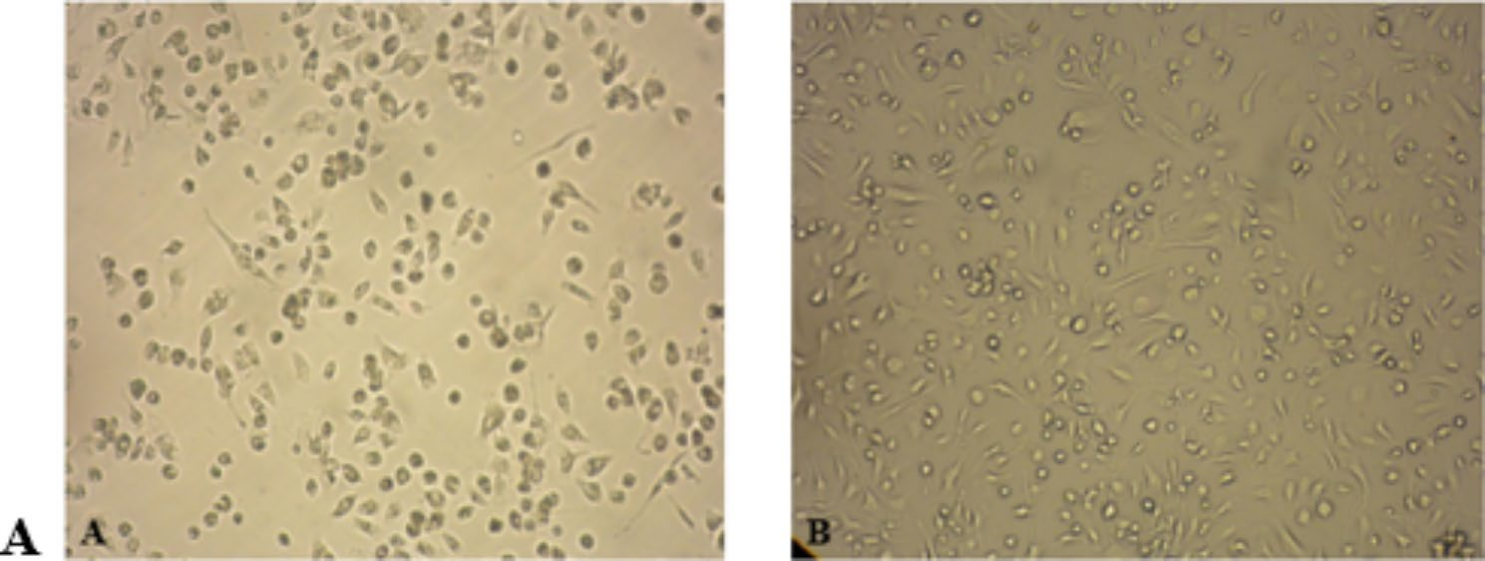
MSCs growth under cell culture conditions. (**A**) 3 days after cell culture, (**B**) seven days after cell culture (40X)

The BALB/c mice were used to isolate MSCs. The cells were kept in monolayer cultures in DMEM with 15% FBS supplement and grown at 37 °C in a humid environment with 5% CO2 (Fig. 1).

**Fig. 2:**
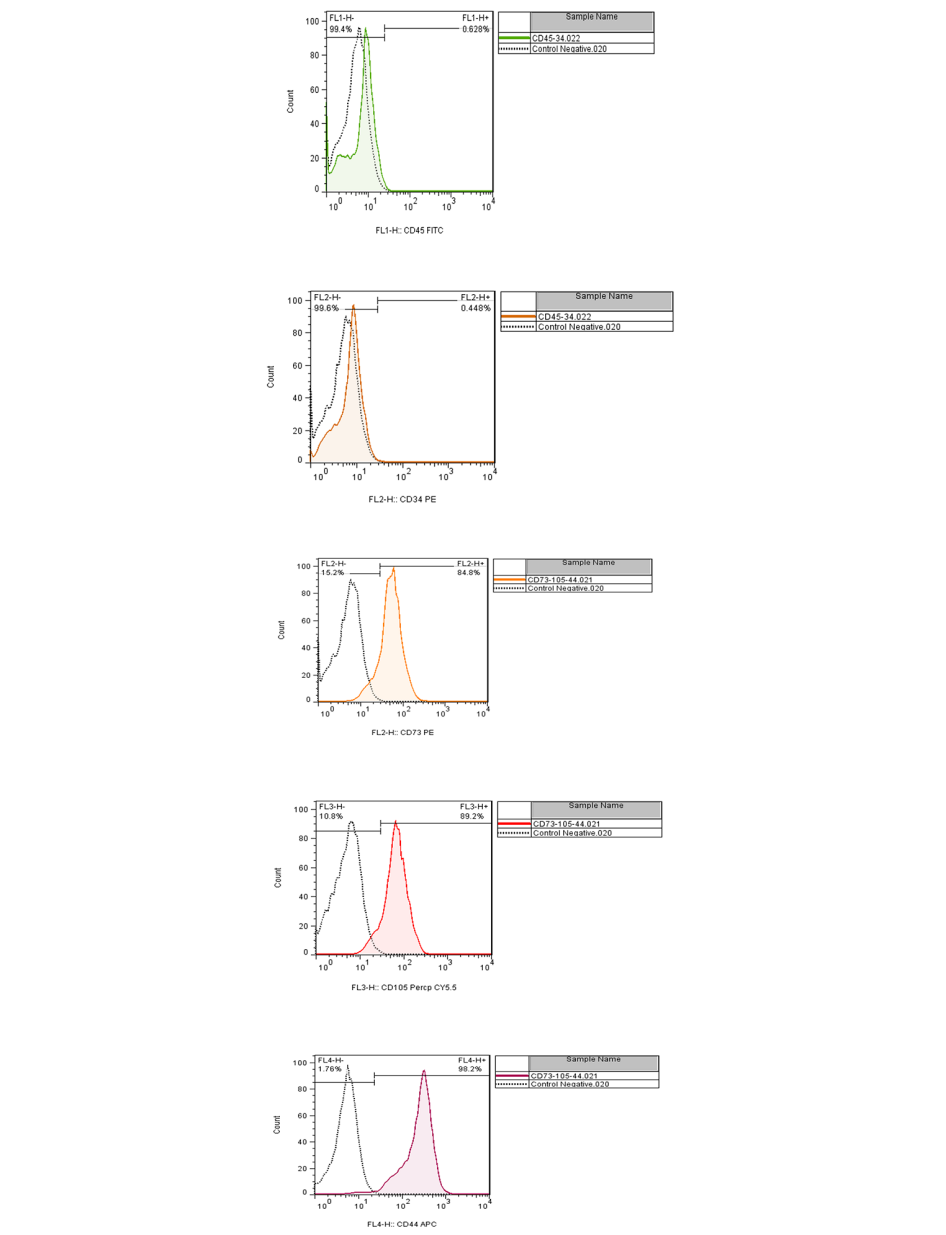
MSCs expressed CD44, CD73, and CD105 but not CD45 and CD34

In passage three, the MSCs were tested for the surface expression of a minimum panel to describe MSCs. In summary, the MSCs were stained with the fluorescently tagged monoclonal antibody and kept in the dark at four°C for 30 min. The labeled cells were then washed three times with wash buffer, resuspended in PBS, and flow cytometry was performed (Fig. 2).

### Cell cytotoxicity (MTT assay)

**Fig. 3:**
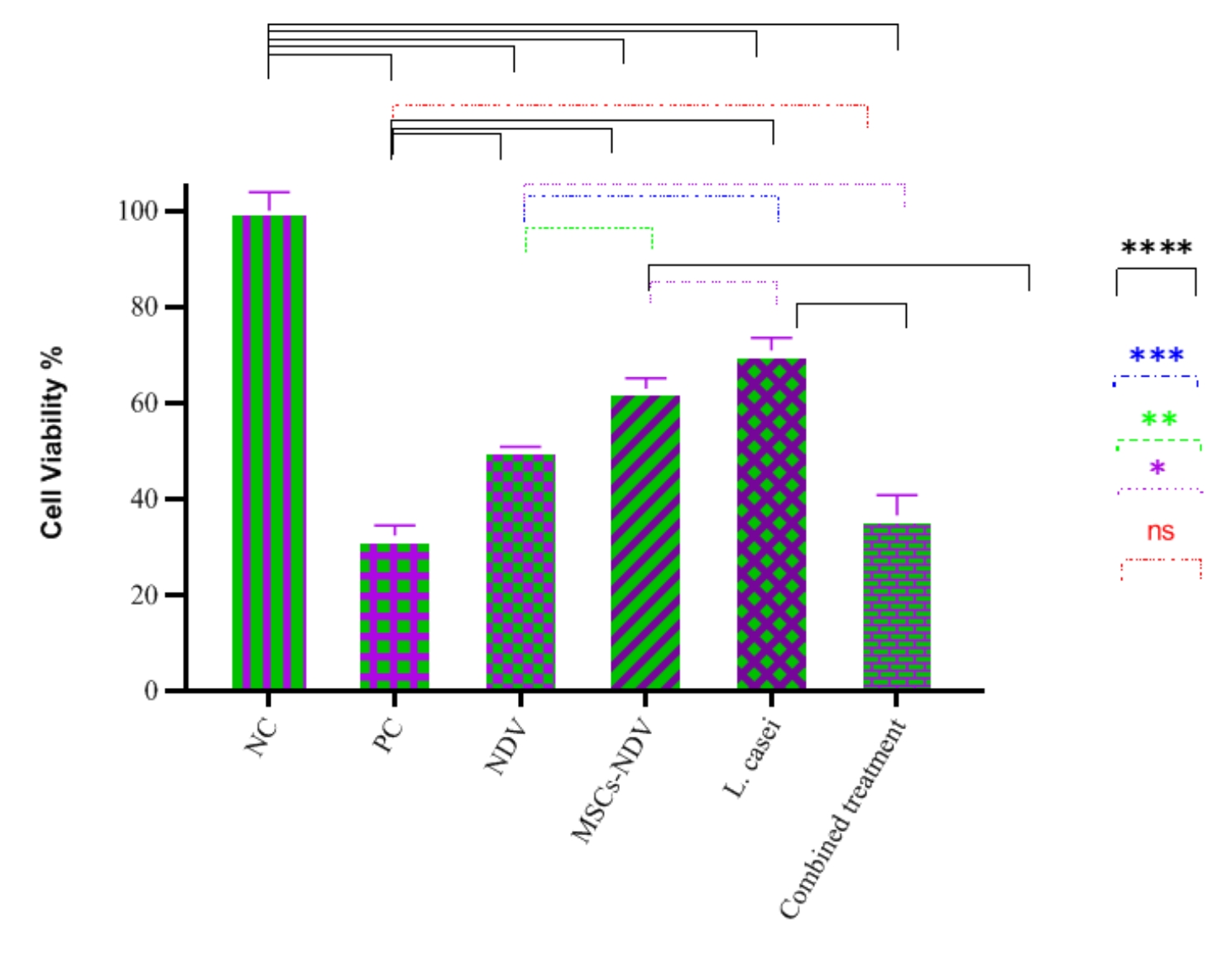
The effect of single or combined treatment on cell viability of the CT26 cell line (* indicated significance at the *P* < 0.05 level, ** indicated significance at the *P* < 0.01 level, *** indicated significance at the *P* < 0.001 level, **** indicated significance at the *P* < 0.0001 level)

As shown in Fig. 3, all treatment groups significantly decreased cell viability compared to the NC group. The PC group and the combined treatment group showed the lowest survival rate compared to the NC group, and there was no significant difference between the PC group, and the combined treatment group. Also, there was a significant difference between the NDV, MSCs-NDV and L. casei treatment groups. The findings proved that NDV has the most and L. casei has the least cytotoxicity.

### Apoptosis results

**Fig. 4:**
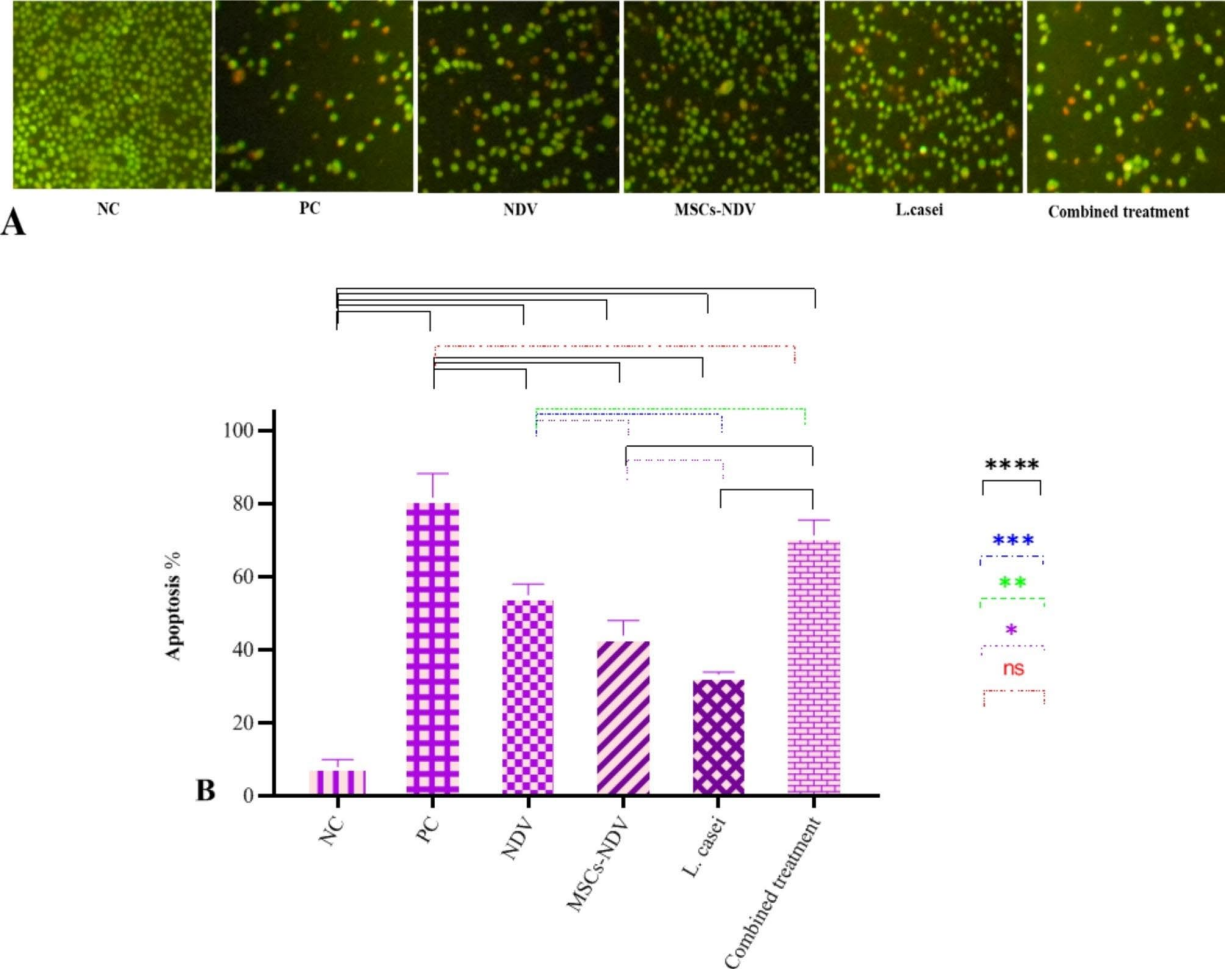
**A**) The assessment of CT26 cells apoptosis by propidium iodide/acridine orange staining (40X). **B**) The effect of single or combined treatment on apoptosis percent of CT26 cell line (* indicated significance at the *P* < 0.05 level, ** indicated significance at the *P* < 0.01 level, *** indicated significance at the *P* < 0.001 level, **** indicated significance at the *P* < 0.0001 level)

According to Fig. 4A, the ability of CT26 cells to survive was assessed using the acridine orange/propidium iodide method. The classification was made based on the chromatin morphology and color of the cell lines. The technique showed that the red cells with no condensed chromatin are necrotic, the condensed chromatin cells are apoptotic, and the green cells with diffused chromatin are alive. Intracellular suicide programs with morphological changes such as cell shrinkage, oxidative stress, coiling, and biochemical reaction resulting in apoptosis are among the effects of cytomorphological alterations in single or combined treatment on CT26 cell lines. As shown in Fig. 4B, all treatment groups significantly increased cell apoptosis compared to the NC group. The PC group and the combined treatment group showed the most apoptosis rate compared to the NC group, and there was no significant difference between the PC group and the combined treatment group. The *L. casei* group showed the most negligible impact on the viability of CT26 cells. In contrast, the NDV group showed the most significant suppression of CT26 cell lines compared to the MSC-NDV, L. casei, and NC groups. The MSCs-NDV and NDV groups’ comparative findings were in perfect accord with the outcomes of the MTT test despite the combined treatment group’s evidence of synergistic benefits. The results revealed that NDV, compared to the MSCs-NDV, has more apoptotic effects, and the reason for this is the late release of the virus from the cell (Fig. 4B).

### ROS production and LDH assay

**Fig. 5:**
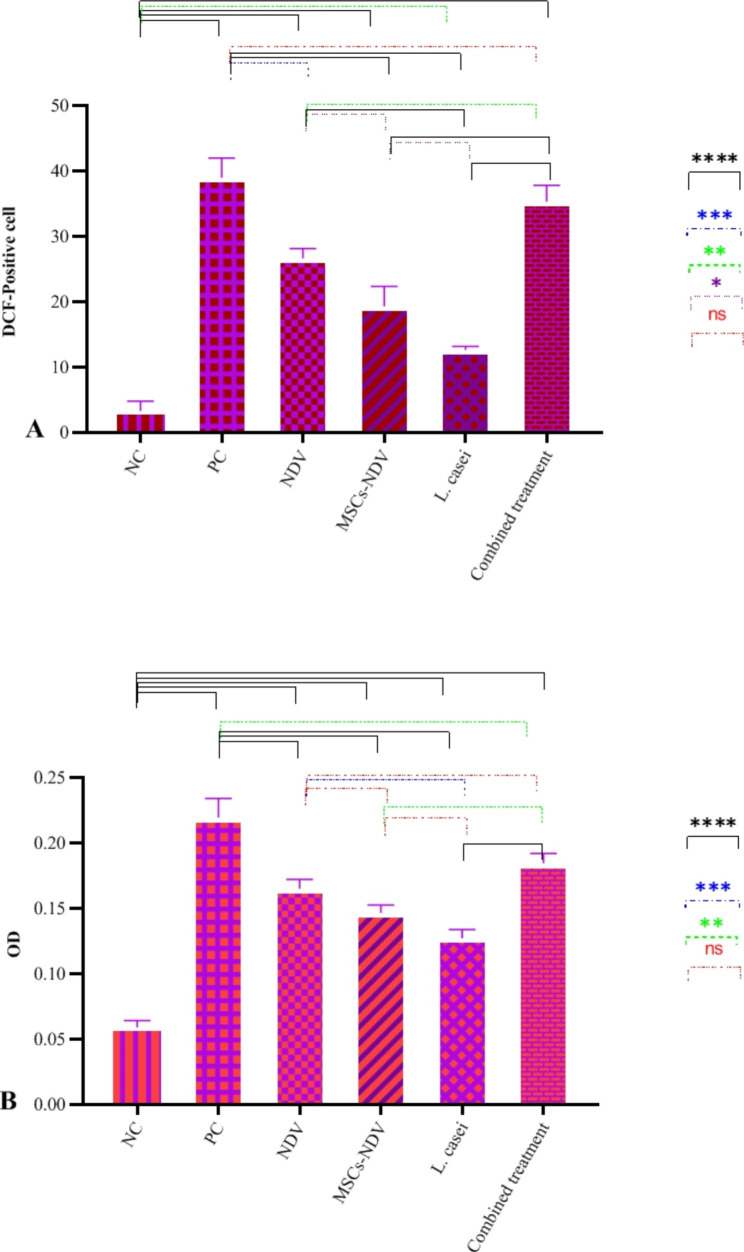
The effect of single or combined treatment on ROS (**A**) and LDH (**B**) production of CT26 cell line (* indicated significance at the *P* < 0.05 level, ** indicated significance at the *P* < 0.01 level, *** indicated significance at the *P* < 0.001 level, **** indicated significance at the *P* < 0.0001 level)

Along with the NDV, MSCs-NDV, *L. casei*, and combined treatments, ROS generation significantly increased in the treated group compared to the NC group (Fig. 5A). The NDV and *L. casei* groups produced the most and least ROS. As shown in Fig. 5B, after treating CT26 cells with NDV, MSCs-NDV, and *L. casei*, LDH activity was evaluated to determine the effects on membrane integrity. The outcomes demonstrated that NDV, MSC-NDV, and L. casei had a more significant impact on the integrity of the cell membrane of CT26 cells than the NC group. The combined treatment group demonstrated synergistic benefits. It did not differ substantially from the PC group while in perfect agreement with the results of the MTT test.

### Caspase assay

**Fig. 6:**
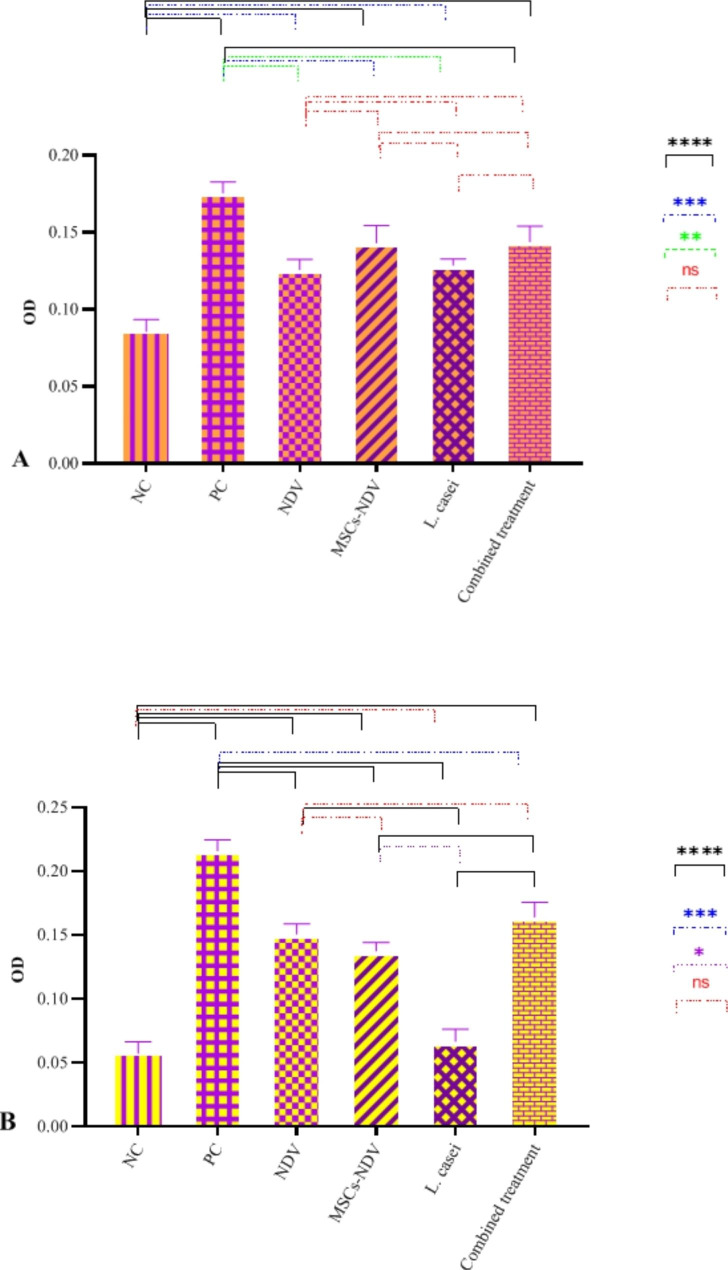
The effect of single or combined treatment on Caspase 8 (**A**) and 9 activity (**B**) of CT26 cell line (* indicated significance at the *P* < 0.05 level, ** indicated significance at the *P* < 0.01 level, *** indicated significance at the *P* < 0.001 level, **** indicated significance at the *P* < 0.0001 level)

Caspase-8 activity levels of the cells treated with NDV, MSCs-NDV, PC, *L. casei*, and combined treatment significantly rise (Fig. 6A). However, Caspase-9 activity levels increased dramatically in NDV, PC, and MSCs-NDV groups (Fig. 6B).

## Discussion

Immune system responses, especially neutralizing antiviral antibodies, inhibit oncolytic viruses from preventing effective infection and reduction of tumor tissue in cancer immunotherapy [37]. Studies of cancer have recently concentrated on using multi-agent therapies, particularly therapeutic agents with synergistic effects, to promote the effectiveness of cancer treatments and overcome the resistance of cancer cells to a particular system [38]. The current study used MSCs as virus carriers to investigate whether the free virus has more cytopathic effects in cell culture conditions, indicating the late release of the virus from the MSCs. The current study used oncolytic bacteria as probiotics to evaluate the synergistic effects. The finding confirmed that the NDV contained in the MSCs has fewer anti-cancer effects than the free NDV because of its sluggish release from the MSCs The animal model results also demonstrated that MSCs harboring oncolytic virus had more substantial anti-cancer effects when L. casei probiotic extract was used concurrently. NDV showed a good safety profile, selective oncolysis, and cancer cell replication among oncolytic virus immunotherapies [39]. Keshavarz et al. (2020) reported that oncolytic NDV could be a particular anticancer potential by inducing autophagic cell death through reactive oxygen species production and activating early apoptotic pathways [40]. Du et al. (2017) showed that MSCs as cell carriers for the herpes simplex virus are a unique and promising strategy for getting around obstacles and enhancing the effector function of oncolytic virotherapy in a tumor microenvironment [41]. Also, numerous studies evaluated the effectiveness of MSCs harboring oncolytic viruses for the cancer treatment [42]. Keshavarz et al. (2020) showed that MSCs harboring oncolytic NDV immunotherapy by triggering splenic Th1 immune responses and death in the tumor microenvironment could be a valuable strategy [33]. Banijamali et al. (2018) showed that high nitric oxide synthase secretion levels and reovirus replication caused apoptosis 48 h after infection. Therefore, maximizing the virus’s duration of MSCs replication makes targeted viral delivery to tumor locations possible and results in the death of cancer cells [43]. Song et al. (2011) reported that one of the fundamental qualities of MSCs is their capacity to implant in tumor tissue, which depends on numerous cytokine receptors, including CXCR4 and matrix metalloproteinase-2 (MMP-2) [44]. Hamada et al. (2007) showed that the carrier cells were used to shield oncolytic viruses from antiviral immune reactions. Also, adenovirus-loaded MSCs can effectively induce antitumoral CTL and antiviral activities using a syngeneic ovarian tumor model [45]. Menstrual blood-derived mesenchymal stem cells (MenSCs) could be used as a delivery system for an oncolytic adenovirus to boost antitumor responses when T and NK cells are activated [46]. Therefore, evidence shows that MSCs as the NDV carrier and 72 h as the time frame is suitable. Numerous *Lactobacillus* strains have strong anti-tumor properties in animal models. *L. casei* increases systemic immune responses in animal models of colon cancer and decreases tumor induction [47]. Also, it causes a cellular immune response that reduces tumor growth and enhances systemic immunological responses in mice that change T-cell activities [48]. Cancer cell lines are inhibited by soluble polysaccharide components from *Lactobacilli* strains, and peptidoglycan produced from cell walls possesses anti-cancer properties [49]. Haghighi et al. showed that combined therapy using *L. casei* and -GalCer is a successful cervical cancer treatment in mouse models [34]. Jafari et al., in a rat model study (2017), demonstrated that the combination immunotherapy using heated 4T1 cells and heated *L. casei* conferred favorable outcomes in breast cancer [50]. In the current study, results showed that NDV, MSCs-NDV, and *L. casei* in alone or combined treatment significantly increased apoptosis percent, LDH, and ROS production compared with the control group. Additionally, the current study’s findings demonstrated that *L. casei* primarily stimulates the extrinsic pathway while NDV therapy promotes apoptosis by activating both intrinsic and extrinsic apoptosis pathways. Extrinsic death-receptor-dependent and intrinsic mitochondrial-dependent apoptosis are the two primary mechanisms that cause apoptosis in mammalian cells. Caspases activation is related to the triggering of cell death in each of these apoptotic pathways [51]. In most studies, the extrinsic death-receptor-dependent pathway is represented by caspase 8, while Caspase-9 represents the intrinsic mitochondrial-dependent mechanism [36]. ROS are mainly formed in mitochondria and are central contributors to oxidative stress and cell death. The high levels of ROS lead to cell death, enhancing the cellular apoptotic pathway [40]. LDH is rapidly released into the cell culture supernatant when the plasma membrane is damaged, a critical feature of cells undergoing apoptosis, necrosis, and other forms of cellular damage [36]. Therefore, the three mentioned variables are aligned and can be considered in the design of treatment methods. NDV is one of the OVs that has been studied comprehensively in the mechanism of apoptosis. NDV-mediated induction of apoptosis includes the activation of endoplasmic reticulum stress and intrinsic and extrinsic apoptotic pathway [52]. Evidence shows that the apoptosis that NDV causes in HeLa cells is mediated mainly by activating the Caspase and TNF-related apoptosis-inducing ligand (TRAIL) pathways [53]. However, the apoptosis that NDV causes in TC1 cells is mostly mediated by mitochondrial pathways [40]. The present study proved that the NDV induces apoptosis in CT26 cells through both pathways. Tiptiri-Kourpeti et al. (2016) showed that *L. casei*-driven up-regulation of the TNF-related apoptosis-inducing ligand TRAIL (extrinsic pathway) and down-regulation of survivin accompanied the inhibition of CRC proliferation. These results show that this probiotic *L. casei* strain has positive tumor-inhibitory, anti-proliferative, and pro-apoptotic properties [54]. Lactic acid bacteria can affect the regulation of apoptosis via intrinsic and extrinsic pathways that are potentially critical mechanisms in preventing colorectal cancer [55]. The current study showed that *L. casei* and NDV boost cancer cells’ production of ROS and LDH. Also, NDV and *L. casei* exhibit synergistic effects in all assessments of their anti-cancer effects.

## Conclusions

The results showed that the MSCs carrier is an effective strategy for delivering oncolytic NDV since it effectively increases ROS and LDH production and triggers the creation of Caspase-9 and 8. Also, the combination of MSCs-NDV and L. casei extract amplifies their anti-cancer actions and makes them two immunotherapeutic agents with synergistic benefits.

## Data Availability

Not applicable.
